# Use of thiacalix[4]arene C-1193 for a directed influence on the functional activity of mitochondria and simulation of this process using a Petri nets

**DOI:** 10.5114/bta.2024.135643

**Published:** 2024-03-29

**Authors:** Hanna Danylovych, Yurii Danylovych, Alexander Chunikhin, Sergiy Cherenok, Vitaly Kalchenko, Sergiy Kosterin

**Affiliations:** 1 Palladin Institute of Biochemistry, National Academy of Science of Ukraine, Kyiv, Ukraine; 2 Institute of Organic Chemistry, National Academy of Science of Ukraine, Kyiv, Ukraine

**Keywords:** mitochondria, thiacalix[4]arenes, Petri nets, calcium, reactive nitrogen and oxygen species

## Abstract

In molecular biological studies, considerable attention is paid to macrocyclic nanoscale compounds known as calix[4]arenes. An imperative concern in biochemical membranology and molecular biotechnology is the exploration of effectors capable of modifying the intensity of redox reactions within the inner mitochondrial membrane and influencing the activity of its Ca^2+^ transport systems. The simulation model development is relevant to formalize and generalize the experimental data and assess the conformity of experimental results with theoretical predictions. Experiments were carried out on a suspension of isolated rat myometrial mitochondria. The synthesized thiacalix[4]arene C-1193, containing four sulfur atoms, was employed. Demonstrations of time-dependent and concentration-dependent (0.01–10 μM) inhibition of Ca^2+^ accumulation and reactive oxygen species (ROS) formation by mitochondria in the presence of C-1193 were observed. While C-1193 inhibited the oxidation of NADH and FADH_2_, it did not induce mitochondrial swelling. The thiacalix[4]arene also inhibited the synthesis of nitric oxide, with a Ki of 5.5 ± 1.7 nM, positioning it as a high-affinity blocker of endogenous NO generation in mitochondria. These results are the basis for the possible application of the synthesized thiacalix[4]arene as a tool in researching biochemical processes in mitochondria. A simulation model employing functional hybrid Petri nets was developed, reproducing the functional activity of mitochondria, including simultaneous NADH oxidation, ROS formation, NO synthesis, and Ca^2+^ accumulation. The derived equations formalize and describe the time dependencies of the listed processes in the medium under the influence of thiacalix[4]arene C-1193.

## Introduction

The disruption of the chain of biochemical and physicochemical processes, collectively contributing to the electro(pharmaco)mechanical coupling phenomenon, forms the basis for the occurrence of various pathologies related to uterine contractile function (including hypotonus and hypertonus, preterm labor, spontaneous abortions, etc.). The regulation of smooth muscle contractile activity is based on changes in cytosolic Ca^2+^ concentration. Substances that modulate the contractile function of myocytes target the Ca^2+^-transport systems of subcellular structures, particularly mitochondria (Wray and Prendergast, 2019). Simultaneously, mitochondrial dysfunction underlies numerous pathological processes, ranging from cardiovascular disorders to the onset of tumors (Alston et al., 2017). Therefore, the search for non-toxic and selective substances that could prevent the development of mitochondrial dysfunction is currently relevant.

In contemporary molecular biological studies, considerable attention is paid to macrocyclic nanoscale compounds known as calixarenes, with the macrocycle cavity exceeding 1 nm^3^ in size. The advantages of calixarenes include their easy synthesis and low toxicity (Pan et al., 2021). Notably, specific calix[4]arenes have demonstrated high-affinity modulation of the activity of various cation transport systems within subcellular membrane structures of uterine smooth muscle (Veklich, 2016; Danylovych et al., 2018). This highlights their potential as modulators of Ca^2+^ transport in mitochondria and the overall functional activity of these organelles. The creation and maintenance of the transmembrane electric potential on the inner membrane of mitochondria, facilitated by the oxidation of organic substrates and the functional activity of the electron transport chain are two of the key links in the organelles' functioning as a Ca^2+^-accumulating system (Anderson et al., 2019; Cao et al., 2019; Alevriadou et al., 2021). The urgent challenge in biochemical membranology and molecular biotechnology is the exploration of effectors capable of modifying the intensity of redox reactions in the inner mitochondrial membrane and influencing the activity of its Ca^2+^ transport systems.

As inherently hydrophobic compounds, calix[4]arenes are able to penetrate cells and interact with mitochondria. Spectrofluorimetry and laser confocal microscopy using specific fluorescent probes and the phenomenon of autofluorescence of calix[4]arenes demonstrated that these compounds (for example C-956) are adsorbed by the surface of the plasma membrane and permeate the myoplasm, interacting with mitochondria (Danylovych et al., 2018). This suggests the potential of calix[4]-arenes to modulate Ca^2+^ transport in mitochondria, instigate the synthesis of reactive nitrogen and oxygen species, and function in the electron transport chain, which is the biochemical basis of the directed influence on the activity of smooth muscles. The thiacalix[4]arene C-1193 (R,S-5,17-bis(dihydroxyphosphonylmethylol)-25,27-dibutoxythiacalix[4]arene) emerges as a promising compound for effectively modulating mitochondrial function.

In previous studies, we demonstrated nitric oxide production in uterine myocytes using the NO-selective fluorescent probe DAF-FM-DA and laser confocal microscopy (Danylovych et al., 2019). Employing DAF-FM and the mitochondria-specific probe MitoTracker Orange CM-H2TMRos colocalization methodology established that mitochondria serve as a powerful source of NO synthesis in myocytes, with the efficiency of nitric oxide formation depending on the functional activity of the electron transport chain (Danylovych et al., 2019). Extensive investigations into the biochemical mechanisms of NO synthesis by these subcellular structures were also conducted using the DAF-FM-DA probe and flow cytometry on isolated rat myometrium mitochondria (Danylovych et al., 2019). Our data underscores the pivotal regulatory role of nitric oxide concerning Ca^2+^ transport systems in the inner mitochondrial membrane and the functional activity of the electron transport chain. Consequently, the search for effective exogenous modulators of NO formation is imperative for precisely influencing these processes.

The formalization and generalization of experimental results and the search for correspondence between theoretical predictions and real-world experimental data, necessitate the utilization of simulation modeling. A modern variant of this approach is the use of a mathematical apparatus namely functional hybrid Petri nets, to model the dynamic states of systems. This methodology affords structural mapping, quantitative analysis, and the ability to consider activating/inhibiting effects (Formanowicz et al., 2017; Cherdal et al., 2018a, 2018b; Gupta et al., 2021). The creation of an adequate model optimizes experimental procedures in terms of time and reagent/laboratory animal costs but also facilitates the analysis of process dynamics. Furthermore, it enables the comparison of experimental results with theoretical calculations under varying conditions in the incubation medium.

Hybrid functional Petri nets offer several advantages as a modeling tool, as highlighted by Wingender (2011):

1. The possibility of structural display of the states of the modeled system and the processes occurring in the system.
2. Quantitative modeling of states and processes of three types at the same time: discrete, continuous, and associative (generic).
3. The ability to take into account activating, inhibitory, and catalytic effects with the help of connections of a special type.

In an effort to leverage these advantages, we applied the methodology of hybrid Petri nets to evaluate changes in biophysicochemical parameters in the functional activity of mitochondria under the influence of thiacalix[4]arene C-1193. The objective was to extrapolate and generalize real experimental results to create a model with a predictive function.

In order to establish the biochemical regularities of the C-1193 compound’s impact on mitochondrial function, the objectives of the study were as follows: investigate its influence on the transport of Ca^2+^, the efficiency of adenine nucleotide oxidation, and the generation of reactive nitrogen and oxygen species.

Conduct simulation modeling of processes, including changes in Ca^2+^ accumulation, intrinsic fluorescence of adenine nucleotides in mitochondria, and the intensity of reactive nitrogen and oxygen species generation, under the conditions of thiacalix[4]arene C-1193 exposure.

## Methods and materials

### Animals

Experiments were conducted on nonpregnant white wild-type female rats with a weight range of 150–180 g (32 animals). All procedures involving animals adhered to the guidelines outlined in the European Convention for the Protection of Vertebrate Animals used for Experimental and other Scientific Purposes (International Convention, Strasbourg, 1986), and the Law of Ukraine *On the protection of animals from cruelty*. The rats were anesthetized using chloroform inhalation and subsequently euthanized by decapitation.

### Isolation of mitochondria from the smooth muscle of the uterus (myometrium) of non-pregnant rats

A preparation of isolated mitochondria was obtained from rat myometrium were prepared using a standard approach involving differential centrifugation (Danylovych et al., 2022). The protein content of the resulting fraction was determined according to Bradford M.M. (Bradford, 1976).

### Examining of the content of ionized Ca^2+^ in mitochondria using spectrofluorometry

Mitochondria were loaded with the Ca^2+^-sensitive fluorescent probe Fluo-4 AM (λ_ex_ = 490 nm, λ_fl_ = 525 nm) at a concentration of 2 μM. This process occurred in a medium comprising 10 mM Hepes (pH 7.4, 37˚C), 250 mM sucrose, 0.1% bovine serum albumin, and 0.02% Pluronic F-127 for 30 min at 37˚C. Investigations into changes in the content of ionized Ca in the matrix of mitochondria were conducted using a Quanta Master 40 PTI spectrofluorimeter (Canada) with FelixGX 4.1.0.3096 software. The Ca^2+^ accumulation process occurred in a medium with the following composition (mM): 20 Hepes (pH 7.4, 37˚C), 250 sucrose, 2 K^+^-phosphate buffer (pH 7.4, 37˚C), 3 MgCl_2_, 3 ATP, and 5 sodium succinate. The Ca^2+^ concentration was maintained at 80 μM. For the study of ΔpH-dependent transport of Ca^2+^ from mitochondria, energy-dependent Ca^2+^ accumulation was performed for 5 min. Subsequently, a 100 μl aliquot of the suspension was diluted in a medium for Ca^2+^ release (2 ml) with the following composition (mM): 20 Hepes (pH 6.5, 37˚C), 250 sucrose, 2 K^+^-phosphate buffer (pH 6.5, 37˚C), 5 sodium succinate, and 0.005 cyclosporin A.

### Study of NO biosynthesis by isolated mitochondria using DAF-FM-DA and spectrofluorometry

The study of nitric oxide synthesis by mitochondria was carried out using the DAF-FM-DA probe (λ_ex_ = 488 nm, λ_fl_ = 525 nm) on a Quanta Master 40 PTI spectrofluorimeter. The loading of the probe at a concentration of 5 μM was carried out in a medium comprising 10 mM Hepes (pH 7.4, 25˚C), 250 mM sucrose, 0.1% bovine serum albumin, and 0.02% Pluronic F-127 for 30 min at 25˚C. The incubation medium’s composition (mM) included 20 Hepes (pH 7.4, 37˚C), 2 K^+^-phosphate buffer (pH 7.4, 37˚C), 125 KCl, 25 NaCl, 5 pyruvate, 5 succinate, 0.01 NADPH, 0.01 tetrahydrobiopterin, 0.05 L-arginine, 0.1 Ca^2+^, with the mitochondrial fraction containing 15–20 μg of protein. The maximum incubation time was set at 30 min.

To determine the apparent inhibition constant (*K*_i_), calculations were calculated in Hill coordinates (Keleti, 1986): {− lg[(*F*_max_ – *F* )/*F* ]; − lg[C-1193]}, where *F*_max_ represents the fluorescence (DAF-FM, relative units) in the absence of thiacalix[4]arene, and *F* is the fluorescence at the respective thiacalix[4]arene concentrations. Curves with a correlation coefficient *R* 2 > 0.9 were taken into account.

### Detection of NADH and FAD fluorescence in mitochondria using spectrofluorometry

The alterations in the relative fluorescence values of adenine nucleotides NADH (λ_ex_ = 350 nm, λ_fl_ = 450 nm) and FAD (λ_ex_ = 450 nm, λ_fl_ = 533 nm) within isolated myometrial mitochondria were recorded using a Quanta Master 40 PTI spectrofluorimeter. The study was carried out in a medium characterized by the following composition (mM): 20 Hepes (pH 7.4, 37˚C), 2 K^+^-phosphate buffer (pH 7.4, 37˚C), 125 KCl, 25 NaCl, 5 pyruvate, and 5 succinate. The protein content in the mitochondrial fraction was maintained at 100 μg.

### Detection of formation of reactive oxygen species in mitochondria using flow cytometry

The ROS formation (changes in DCF fluorescence) in isolated mitochondria was conducted using the flow cytometry method on a COULTER EPICS XL^TM^ flow cytometer (Beckman Coulter, USA), λ_ex_ = 488 nm, λ_fl_ = 515 nm (Fl1 channel). The mitochondria were loaded with the fluorescent probe DCF-DA at a concentration of 25 μM. The loading process occurred in a medium comprising 10 mM Hepes (pH 7.4, 37˚C), 250 mM sucrose, 0.1% bovine serum albumin, and 0.02% Pluronic F-127 for 30 min at 25˚C. The incubation medium (2 ml) had the following composition (mM): 20 Hepes (pH 7.4, 25˚C), 2 K^+^-phosphate buffer (pH 7.4, 25˚C), 125 KCl, 25 NaCl, 5 pyruvate, and 5 succinate. The reaction was initiated by adding a 20 μl aliquot of 5 mM pyruvate + 5 mM succinate. The protein content in the mitochondrial fraction used was in the range of 20–25 μg.

### Estimation of mitochondrial hydrodynamic diameter

The distribution function of the hydrodynamic diameter (characteristic size) of mitochondria was determined through photon correlation spectroscopy (Merkus, 2009). The ZetaSizer-3 device (Malvern Instruments, Great Britain) with the Multi8 computing correlator type 7032 ce, which is equipped with a helium–neon LGH-111 laser with a wavelength of 633 nm and a power of 25 mW was used. Laser radiation scattered from the mitochondrial suspension was registered for 10 min at a temperature of 24˚C and a scattering angle of 90˚, with data collected every 1 min. The auto-correlation function was processed using the standard computer program PCS-Sizemode v 1.61. The incubation medium (1 ml) used for this analysis had the following composition (mM): 20 Hepes (pH 7.4, 25˚C), 2 K^+^-phosphate buffer (pH 7.4, 25˚C), 125 KCl, 25 NaCl, 5 pyruvate, and 5 succinate. The protein content in the mitochondrial fraction was 50 μg.

The solutions were prepared in bidistilled water with a specific electrical conductivity not exceeding 2.0 μcm/cmc, and the electrical conductivity was measured using a conductometer OK-102/1 (Hungary).

### Statistical methods

The data is expressed as means ± SE based on the numbers of determinations (*n* = 4–7). Statistical comparisons between datasets from fluorometric experiments were analyzed using unpaired Student’s *t*-tests conducted in Microsoft Excel.

### Simulation modeling

For modeling purposes, we opted for the Cell Illustrator v.3 software environment (Human Genome Center, University of Tokyo, Japan). This platform utilizes hybrid functional Petri nets as its fundamental apparatus. A Petri net is a directed bipartite graph encompassing two types of vertices (Table 1): places and transitions, interconnected by arrows (arcs) that reflect the network’s structure. Typically, positions characterize objects, elements, and resources within the modeled system, while transitions represent processes occurring in the system and the logical conditions for their implementation.

**Table 1:** The main structural elements of the hybrid functional Petri net (explanation in the text)

Type	Places	Transitions	Label	Arcs
Discrete	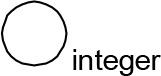 Discrete place	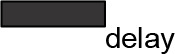 Discrete transition	Normal	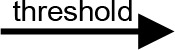 Normal arc
Continuous	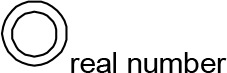 Continuous place	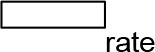 Continuous transition	Test	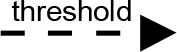 Test arc
Generic	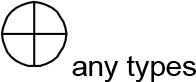 Generic place	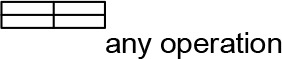 Generic transition	Inhibitory	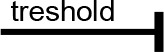 Inhibitory arc

### Synthesis of thiacalix[4]aren-bis-hydroxymethylphosphonic asids C-1193

The reactions were carried out in anhydrous solvents. Tris(trimethylsilyl)phosphite and diformyldibutoxythiacalixarene were synthesized following literature methods (Yanga et al., 2004; Yang et al., 2012; Cherenok et al., 2012). Specifically, a solution of diformyldibutoxythiacalixarene (1 mmol) in dry methylene chloride (20 ml) was prepared, and tris(trimethylsilyl)phosphite (5 mmol) was added at room temperature. The resulting mixture was stirred for 15 h. Residual methylene chloride and silyl phosphite were removed under reduced pressure, and the remaining product was subjected to a vacuum of 0.1 mm Hg at 50˚C for 2 h. Subsequently, wet methanol (30 ml) was introduced to the obtained silyl derivative of thiacalixarene, and the reaction mixture was stirred for 2 h at 40˚C. The solvent was evaporated under reduced pressure, and the residue was further subjected to a vacuum of 0.1 mm Hg at 50˚C for 2 h. C-1193 acid was dissolved in methanol (10 ml) and diluted with water (10 ml). The resulting precipitate was filtered, dried under a vacuum of 0.1 mm Hg at room temperature for 5 h.

### Characteristics of the obtained compound C-1193

The nuclear magnetic resonance (NMR) spectra were registered on a Varian VXR-400 spectrometer operating a 399.987 MHz (^1^H), 150.8 MHz (^13^C), using TMS as reference. The ^31^P NMR spectra were recorded on a Varian VXR-400 spectrometer operating a 162 MHz using 85% H_3_PO_4_ as a reference. Melting points were measured on a Boёtius heating block and were uncorrected.

Colorless crystals 26,28-Dihydroxy-25,27-dibutoxythiacalix[4]arene-5,17-bis("-hydroxymethylphosphonic acid): yield 92%., m.p. 221–224˚E. ^1^H NMR (DMSO), δ: 1.03 (t, 6H, *J* 7.5 Hz, CH_2_C***H***_3_), 1.58 (m, 4H, CH_2_C***H***_2_CH_3_) 1.88 (m, 4H, C***H***_2_CH_2_CH_3_), 4.4 (t, 4H, *J* 6.2 Hz, OC***H***_2_), 4.68 (d, 2H, *J* 12.5 Hz,PC***H***), 6.66 (t, 2H, *J* 8.0 Hz, p-Ar***H***), 6.94, 7.00 (two d, 4H, *J* 8.0 Hz, M-Ar***H*** ), 7.68 (s, 2H, O***H***), 7.70, 7.75 (two s, 2=+2H, M-Ar***H***). ^31^P NMR (DMSO), δ 18.1. ^13^C NMR (DMSO), δ14.24, 19.07, 32.06, 69.6 (д, *J*_PC_ = 159 Hz), 75.56, 121.27, 121.52, 125.91, 129.34, 132.28, 135.81, 136.16, 156.63, 158.39; Mass (FAB) m/z; 840 [M + H]^+^. Calcd. for C_34_H_49_O_12_P_2_S_4_ 839.16.

The energy minimized conformation of C-1193 was obtained by molecular mechanics PM3 method (software package Hyper Chem, version 8) (http://www.hyper.com/Download/Alldownloads/tabid/470/Default.aspx). RMS gradient was 0.01 kcal/mol.

Thiacalix[4]arene C-1193 was used as a solution in dimethylsulfoxide (DMSO). The aliquots were added directly to the incubation medium (the final concentration of DMSO was less than 0.05%).

## Results and discussion

**Fig. 1:**
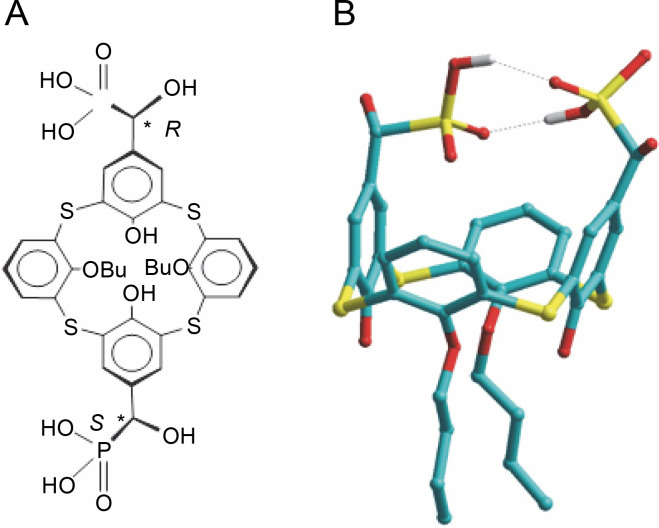
Thiacalix[4]arene C-1193: molecular structure (A) and energy minimized cone conformation (B)

The amphiphilic cone-shaped C-1193, characterized by two hydrophilic α-hydroxymethylphosphonic groups at the macrocyclic upper rim and two lipophilic butyl groups at the lower rim (Fig. 1), exhibited the ability to traverse the plasma membrane, exerting an impact on intracellular compartments, notably mitochondria. However, due to the distinctive composition of protein and lipid components in the inner mitochondrial membrane – particularly the notably high protein content of respiratory chain complexes – the diffusion of compound C-1193 into the matrix was restricted. We posit that its potential effects on the electron transport chain primarily arise from interactions with the outer part of the inner membrane.

**Fig. 2:**
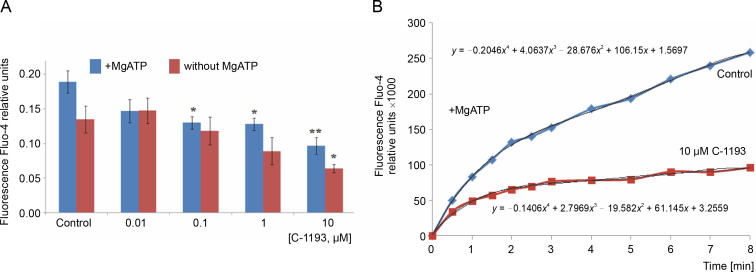
Effect of thiacalix[4]arene C-1193 on Ca^2+^ accumulation (A) in mitochondria;*P < 0.05, ** *P* < 0.01 vs control, *n* = 4–6 (the data is presented as mean ± SE); (B) an example of the dynamics of Fluo-4 fluorescence changes in the control and under the action of 10 μM C-1193, which were used to calculate analytical dependencies in the model (the data of a typical experiment with polynomial curves and corresponding equations are presented)

The systems responsible for maintaining Ca^2+^ homeostasis in organelles are located in the inner mitochondrial membrane, specifically structures facilitating energydependent Ca^2+^ accumulation into the matrix through an electrophoretic mechanism. The presence of thiacalix[4]-arene C-1193 led to a time-dependent and concentration-dependent (0.01–10 μM) inhibition of Ca^2+^ accumulation by mitochondria (Fig. 2).

This effect was observed in both scenarios: the accumulation of Ca^2+^ by mitochondria, whose function depended solely on the presence of oxidation substrates and under conditions of additional energization through the introduction of exogenous Mg-ATP^2−^. In the latter case, the reverse function of ATP synthetase, contributing to the creation of a proton gradient on the inner membrane, provided an additional driving force for the energy-dependent accumulation of Ca ions. The effect of C-1193 on the energy-dependent accumulation of Ca^2+^ by mitochondria was specific. In parallel studies, this thia-calix[4]arene exhibited no discernible effect on the H^+^/Ca^2+^ exchanger, another transport system also localized in the inner mitochondrial membrane of uterine myocytes (Danylovych et al., 2022) (graphical data not shown).

The ability of mitochondria to accumulate Ca^2+^ was pivotal for the overall cellular function, given that their ATP production was contingent upon the concentration of this cation in the matrix. Simultaneously, Ca^2+^ overloading of mitochondria served as a trigger for the opening of the permeability transition pore and the development of apoptosis (Bock and Tait, 2020). A reduction in the intensity of Ca^2+^ influx into mitochondria was accompanied by a decrease in the concentration of the cation in the matrix, which could impact the intensity of Ca^2+^-dependent processes. Notably, the functioning of the electron transport chain in the inner mitochondrial membrane, involving Ca^2+^-dependent enzymes such as the pyruvate dehydrogenase complex, α-ketoglutarate dehydrogenase, and isocitrate dehydrogenase, should be emphasized (Gellerich et al., 2010). Furthermore, the synthesis of reactive nitrogen and oxygen species, particularly NO, is stimulated by Ca ions (Giulivi et al., 2006; Traaseth et al., 2004; Matuz-Mares et al., 2022). However, excessive elevation of Ca^2+^ concentration in mitochondria is dangerous and can lead to mitochondrial dysfunction (Anderson et al., 2019). In this context, the inhibition of the energy-dependent transport of Ca^2+^ to mitochondria by C-1193 could be construed as a potential protective effect.

**Fig. 3:**
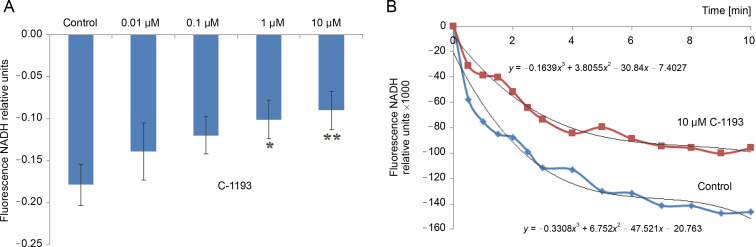
Effect of thiacalix[4]arene C-1193 on NADH oxidation (A) in isolated mitochondria; *P = 0.05, **P < 0.05 vs control, *n* = 5 (the data is presented as mean ± SE); (B) an example of the dynamics of NADH fluorescence changes in the control and under the action of 10 μM C-1193, which were used to calculate analytical dependencies in the model (the data of a typical experiment with polynomial curves and corresponding equations are presented)

**Fig. 4:**
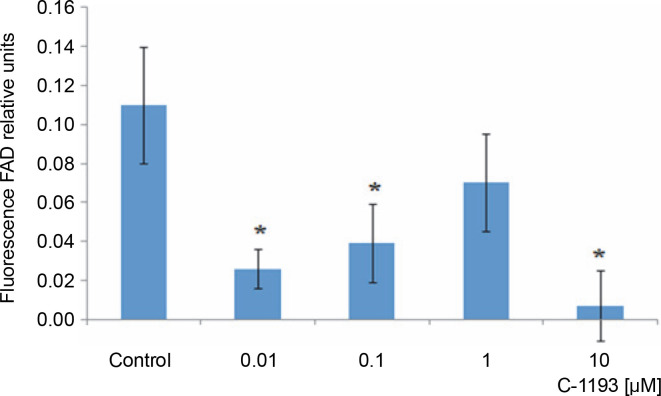
The effect of the studied thiacalix[4]arene on the oxidation of FADH_2_ in mitochondria; *P < 0.05 vs control, *n* = 5 (the data is presented as mean ± SE)

Alterations in the redox state of adenine nucleotides NADH/FADH_2_ serve as indicators of the functionality of the mitochondrial electron transport chain (Kosterin et al., 2005; Heikal, 2010). To energize the mitochondria, pyruvate and succinate (5 mM each) were introduced into the incubation medium. Over time, the fluorescence signal in mitochondria from NADH decreased, while that from FAD increased, indicative of the dynamic activity of the electron transport chain. Thiacalix[4]arene C-1193 exhibited a concentration-dependent inhibition (0.01–10 μM) of NADH oxidation in isolated mitochondria (Fig. 3) and had an inhibitory effect on FADH_2_ oxidation (Fig. 4). Under the experimental conditions (nominal absence of Ca^2+^ in the medium during the recording of the fluorescent signal from NADH/FAD), these effects may be attributed to a direct effect on the electron transport chain. Likely, the inhibition of the functional activity of complex I in the presence of C-1193 underlies this effect. However, it is also plausible that the observed impact of C-1193 may also be associated with the inhibition of exogenous Ca^2+^ entry into mitochondria *in vivo*. The lack of a pronounced concentration dependence of the C-1193 effect on the fluorescence signal from FAD may suggest the complex nature of its impact on the electron transport chain.

The effectiveness of Ca^2+^ transport systems in mitochondria is contingent upon the respiratory chain’s activity and changes in the modulus of the electrical potential of the inner mitochondrial membrane. The potential suppression of the functional activity of respiratory chain complexes, resulting in a decrease in the electric potential on the inner mitochondrial membrane and the intensity of oxidative phosphorylation under the influence of C-1193, could impact both the bioenergetics of mitochondria and the functioning of Ca^2+^ transport systems in their inner membrane. This could elucidate the reduction in Ca^2+^ accumulation by mitochondria in response to C-1193 in our experiments (Fig. 2). However, it is also quite likely that the studied compound exerts a direct effect on the structures of the Ca^2+^-uniporter.

**Fig. 5:**
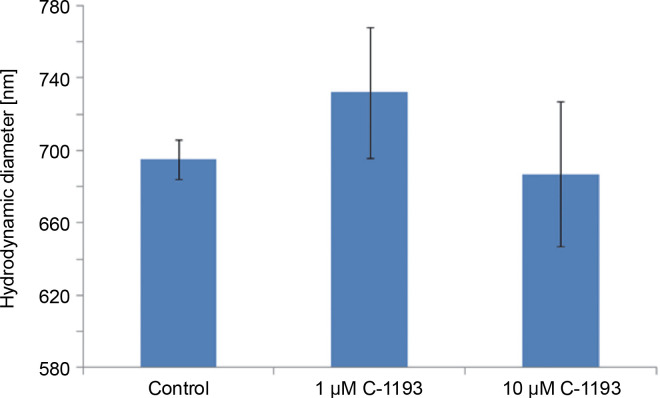
Changes in the hydrodynamic diameter of isolated mitochondria under the action of thiacalix[4]arene C-1193, *n* = 4 (the data is presented as mean ± SE)

Alterations in the functioning of the electron transport chain can manifest in changes in organelle volume (Kaasik et al., 2007). For example, hyperpolarization of the inner membrane leads to a reduction in volume. Photon correlation spectroscopy, an effective method for studying changes in the size of near-spherical particles in solutions (Mercus, 2009), was used in this study. At concentrations of 1 and 10 μM, thiacalix[4]arene C-1193 did not induce any changes in the characteristic dimensions (hydrodynamic diameter) of mitochondria, indicating that it did not cause mitochondrial swelling (Fig. 5). It is noteworthy that, according to photon correlation spectroscopy data, C-1193 at the concentrations used in these experiments did not result in noticeable micelle formation.

Mitochondrial swelling is often a consequence of the disturbance of the osmotic balance between the matrix and the surrounding environment, frequently induced by the opening of the permeability transition pore (Brocard et al., 2003; Kaasik et al., 2007; Nowikovski et al., 2009). This can lead to ruptures in the outer mitochondrial membrane and an increased release of proapoptotic factors into the cytosol. Therefore, significant mitochondrial swelling is indicative of disturbances in their functioning. Our results confirm that thiacalix[4]arene C-1193, at the concentrations used, did not induce mitochondrial dysfunction based on morphological features.

**Fig. 6:**
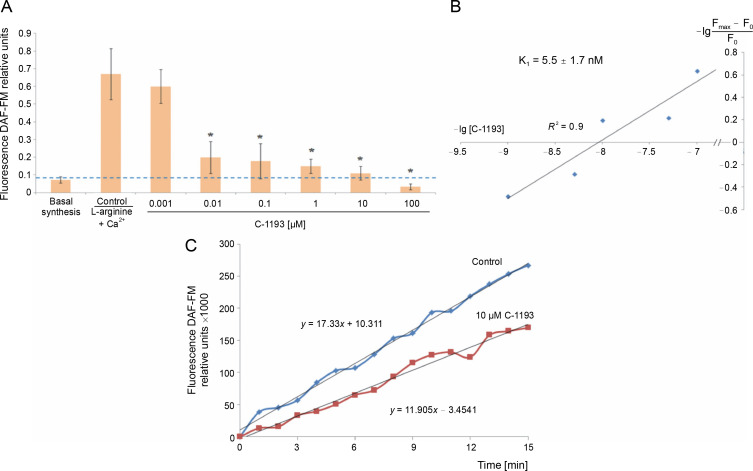
Effect of thiacalix[4]arene C-1193 on Ca^2+^-dependent synthesis of nitric oxide in mitochondria (A), *P < 0.01 vs control, *n* = 7 (the data is presented as mean ± SE); (B) example of inhibition constant (*K*_i_ ) calculation by Hill’s method; (C) an example of the dynamics of DAF-FM fluorescence changes in the control and under the action of 10 μM C-1193, which were used to calculate analytical dependencies inthe model (thedata of a typical experiment with polynomial curves and corresponding equations are presented)

Previously, we established that the synthesis of nitric oxide by myometrial mitochondria is a Ca^2+^-dependent process (Danylovych et al., 2019). The investigated thiacalix[4]arene effectively inhibited the synthesis of nitric oxide by mitochondria in a concentration-dependent manner (0.001–100 μM) (Fig. 6). The inhibition constant calculated in Hill’s coordinates was 5.5 ± 1.7 nM (*n* = 7), making the studied compound a high-affinity blocker of endogenous NO generation. The effect of C-1193 on NO synthesis can be explained both by the inhibition of Ca^2+^ entry into the matrix under the influence of the investigated thiacalix[4]arene and by a direct effect on mitochondrial NO-synthase, which is associated with the inner membrane of mitochondria.

It is currently established that NO regulates the functional activity of mitochondria, particularly endogenously synthesized NO. Nitric oxide affects the functioning of the electron transport chain, reversibly inhibiting cytochrome c oxidase, and controlling the pH value in the matrix (Valdez et al., 2006; Giulivi, 2007; Shiva, 2010). Nitric oxide at low nanomolar concentrations limits the intensity of respiration and oxidative phosphorylation, considered an adaptive physiological response (Giulivi et al., 2006; Brown and Borutaite, 2007; Tengan et al., 2012). NO regulates Ca^2+^ homeostasis in mitochondria and, accordingly, Ca^2+^-dependent processes (Traaseth et al., 2004; Giulivi et al., 2006; Giulivi, 2007). ATP synthesis by mitochondria is cGMP-dependent and regulated by nitric oxide (Moon et al., 2017). Nitric oxide also stimulates mitochondria biogenesis (Tengan et al., 2012; Piantadosi and Suliman, 2012). On the other hand, excessive NO production along with increased formation of superoxide anion in mitochondria leads to significant peroxynitrite generation. Peroxynitrite causes damage to components of the electron transport chain, irreversible depolarization of organelles, and the development of mitochondrial dysfunction. The reaction of NO with O_2_^•−^ is a crucial factor in reducing the bioavailability and physiological activity of nitric oxide in mitochondria. Peroxynitrite induces oxidative damage to mitochondrial proteins, including irreversible inactivation of Mn^2+^-containing superoxide dismutase and matrix aconitase (Tortora et al., 2007; Demicheli et al., 2016). Nitrosative stress compromises the structural and functional properties of lipids and DNA (unrepaired ruptures and other damages), leading to apoptosis or even necrosis (Salem et al., 2009; Brown, 2010; Santos et al., 2011; Litvinova et al., 2015). Therefore, the effective suppression of NO synthesis by the studied thiacalix[4]arene is a prerequisite for its potential use in preventing the development of nitrosative/oxidative stress in mitochondria and the corresponding mitochondrial dysfunction.

**Fig. 7:**
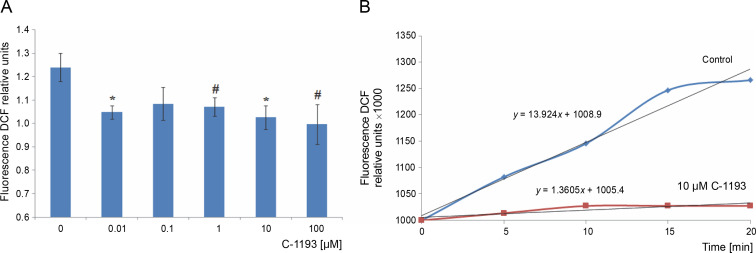
The effect of compound C-1193 on the formation of reactive oxygen species (A) in isolated mitochondria, *P < 0.01, #*P* = 0.05 vs control, *n* = 5 (the data is presented as mean ± SE); (B) an example of the dynamics of DCF fluorescence changes in the control and under the action of 10 μM C-1193, which were used to calculate analytical dependencies in the model (the data of a typical experiment with polynomial curves and corresponding equations are presented)

Mitochondria are considered a source of ROS, which plays signaling and regulatory functions at low concentrations (Dunn et al., 2015; Matuz-Mares et al., 2022). The efficiency of the electron transport chain reflects the level of ROS formation within mitochondria (Chen and Zweier, 2014). Simultaneously, heightened ROS generation in the respiratory chain results in oxidative stress, leading to mitochondrial dysfunction. The fluorescent probe DCF serves as an effective indicator of ROS formation intensity. The investigation revealed that the studied thiacalix[4]arene, contingent upon concentration (0.01–100 μM), inhibited ROS formation in mitochondria (decreased DCF fluorescence) (Fig. 7). DCF detects the production of superoxide anion, hydrogen peroxide, hydroxyl radicals, and peroxynitrite, formed from the reaction of NO and O_2_^•−^, particularly under nitrosative/oxidative stress conditions. The findings indicate that the utilized calix[4]arene does not elicit similar effects.

A reduction in the electron transport chain’s activity, along with the inhibition of Ca^2+^ accumulation in mitochondria and Ca^2+^-dependent nitric oxide synthesis, correlates with the suppression of ROS generation. This suggests a potential protective effect of compound C-1193 on mitochondria (Chen and Zweier, 2014; Dunn et al., 2015; Bock and Tait, 2020).

A simulation model depicting the impact of C-1193 on mitochondrial functioning parameters was developed using Petri nets, based on the aforementioned experimental data.

**Fig. 8:**
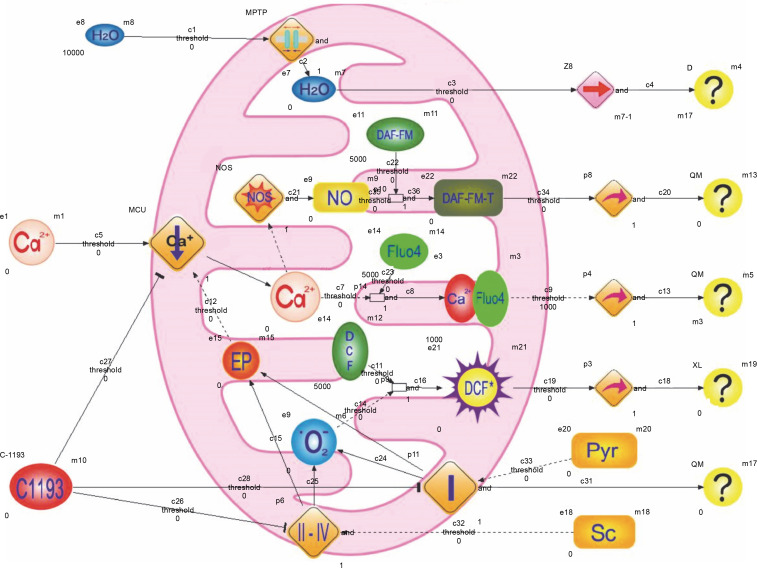
The structure of hybrid functional Petri nets that simulate the effects of E-1193 on changes in the fluorescence of Fluo-4, DAF-FM (DAF-FM-G is a fluorescent triazolofluorescein derivative that is formed due to the interaction of DAF-FM with NO), DCF (DCF^*^ – active oxidized form DCF) and NADH in isolated mitochondria; symbols on the diagram: NO – nitrogen oxide, NOS – NO-synthase, Sc – sodium succinate, Pyr – sodium pyruvate, O_2_^•−^ – superoxide anion, I–IV – electron transport chain complexes, EP – electric potential, the symbol – | | – in the middle of the rhombus – cyclosporine-sensitive mitochondria permeability transition pore (MPTP), arrows: → – activation process, ⊺ – inhibition process

The model outlined below incorporates a significant simplification of the actual scenario, recognizing the inherent idealization of highly intricate biological processes. The model considers components of the incubation medium and the following experimental observations: 1) succinate and pyruvate are introduced into the medium to energize mitochondria; the electron transport chain’s operation induces an electric potential on the inner mitochondrial membrane, serving as the driving force for the electrophoretic accumulation of Ca^2+^ by mitochondria; 2) inhibition of electron transport chain activity, particularly complex I, results in reduced endogenous NADH fluorescence, increased production of reactive oxygen species in mitochondria, and a decline in DCF fluorescence; 3) inhibition of Ca^2+^ accumulation by mitochondria is reflected in diminished Fluo-4 fluorescence; 4) reduction in Ca^2+^ concentration in the matrix hampers Ca^2+^-dependent synthesis of nitric oxide, leading to decreased DAF-FM fluorescence; 5) mitochondrial hydrodynamic diameter growth predominantly occurs due to the permeability transition pore opening, disruption of osmotic balance, water molecule transport into the matrix, and organelle swelling (Fig. 8).

During the modeling process, mathematical equations were derived to formalize the experimental data. Analytical expressions were obtained to describe the dynamics of experimental changes in the relative fluorescence of NADH, DCF*, DAF-FM-T, and Fluo-4. Additionally, calculated values for the electric potential were determined under both control conditions and in the presence of C-1193 at a concentration of 10 μM. The results of measurements depicting changes in the studied parameters over time are illustrated in the corresponding figures. Polynomial approximations ranging from first to fourth degree were applied to fit the experimental curves. All equations remain valid within the approximation interval from 0 to 15 min and were computed based on averaged values obtained from four experiments. For ease of computation, the relative units of fluorescence are multiplied by a coefficient (× 1000).

The dynamics of NADH fluorescence changes in the control (Fig. 3B):

−0.38 *t* ^3^ + 7.44 *t* ^2^ – 49.20 *t*.

Time dependence of NADH fluorescence reduction in the presence of C-1193:

−0.16 *t* ^3^ + 3.81 *t* ^2^ – 30.80 *t*.

Accordingly, the activation speed of the transition (I) in the Petri net (Fig. 8) is:

−1.14 *t*^2^ + 14.88 *t* – 49.20 (in the control)

and −0.48 *t*^2^ + 7.62 *t* – 30.80 (in the presence of C-1193).

The oxidation of adenine nucleotides and the functioning of the electron transport chain are prerequisites for generating an electric potential on the inner mitochondrial membrane. Calculated values for changes in electric potential (EP, mV) were derived from analytical dependencies describing the dynamics of NADH fluorescence changes. The corresponding equations are formulated as follows:

−0.38 *t* ^3^ + 7.44 *t* ^2^ – 49.20 *t* – 40.00 (in the control)

and !0.16 *t* ^3^ + 3.81 *t* ^2^ – 30.80 *t* – 40.00

(in the presence of E-1193).

The equations that reflect the dynamics of changes in DCF*-fluorescence (Fig. 7B) have the following form:

13.9 *t* + 1000 (in the control)

and 1.3 *t* + 1000 (in the presence of C-1193).

Time dependences of Fluo-4 fluorescence changes according to the results of the experiment (Fig. 2B) are described by the equations:

−0.20 *t* ^4^ + 4.06 *t* ^3^ – 28.68 *t* ^2^ + 106.15 *t* + 1.57

(in the control)

and −0.14 *t* ^4^ + 2.80 *t* ^3^ – 19.58 *t* ^2^ + 61.15 *t* + 3.26

(in the presence of C-1193).

Changes in DAF-FM-T-fluorescence according to experimental data up to 15 min of incubation in the mode of the initial rate of NO synthesis (Fig. 6C) satisfy the equation:

17.39 *t* +16.91(in the control)

and 13.12 *t* – 8.23 (in the presence of E-1193).

To determine the efficiency and regularities of NO synthesis, linearized dependencies of Fluo-4 fluorescence changes were examined using a two-component approximation within the time intervals from 0 to 2.5 min and from 2.5 to 15 min (Fig. 2B). In the case of the control condition, the following equations apply:

27.68 *t* – 11.41 (in the interval from 0 to 2.5 min),

20.44 *t* + 116.03 (in the interval from 2.5 to 15 min).

In the presence of C-1193:

12.86 *t* + 1.01 (in the interval from 0 to 2.5 min),

4.17 *t* + 66.43 (in the interval from 2.5 to 15 min).

Analysis of the obtained regularities allows us to put forward two hypotheses.

1. The initial velocity of NO synthesis in the control (V_0_^NO^) is determined by both the initial velocity of Ca^2+^ accumulation in the matrix (V_0_^Ca^) and the initial velocity of mitochondria energization, i.e. the rate of the electric potential growth upon addition of oxidation substrates (V_0_^EP^): V_0_^NO^ = V_0_^Ca^ + V_0_^EP^.
2. In the presence of 10 μM thiacalix[4]arene C-1193, the rate of NO synthesis is determined mainly by the initial velocity of Ca^2+^ accumulation in the matrix. The regulatory influence of mitochondrial energization becomes less significant: V_0_^NO^ ⇒ V_0_^Ca^.

The given model provides an adequate experimental data description of changes in the fluorescence of Fluo-4, DAF-FM, DCF, and NADH in mitochondria, which allows to significantly optimize the time of experimental procedures, reagents, and laboratory animals. In addition, it allows analyzing the dynamics of processes and comparing the consequences of modeling (theoretical predictions) with actual observations.

## Conclusion

The sulfur-containing thiacalix[4]arene C-1193 induces alterations in Ca^2+^ transport activity, effectively suppresses NO synthesis, and exerts a notable modulatory influence on the electron transport chain of myometrial mitochondria. The inhibition of reactive oxygen species formation in mitochondria by the used thiacalix[4]arene and the absence of mitochondrial swelling indicates that the investigated processes do not lead to the development of mitochondrial dysfunction. These results are the basis of the possible application of the selected thiacalix[4]arene as a valuable tool in the research of biochemical processes associated with mitochondria.

The proposed mathematical model, employing the methodology of Petri nets, serves to formalize and generalize experimental data, enabling a prognostic function and facilitating the exploration of correspondence between theoretical predictions and actual results. The equations effectively capture the time characteristics of changes in the fluorescence of Fluo-4, DAF-FM, DCF, and NADH, providing accurate predictions of the intensity under varying conditions, such as changes in the incubation medium.

## Conflict of interest

The authors declare no conflict of interest

## References

[cit0001] Alevriadou B.R., Patel A., Noble M., Ghosh S., Gohil V.M.,. Stathopulos P.B., Madesh M. (2021) Molecular nature and physiological role of the mitochondrial calcium uniporter channel. Am. J. Physiol. Cell Physiol. 320: C465–C482. 33296287 10.1152/ajpcell.00502.2020 PMC8260355

[cit0002] Alston C.L., Rocha M.C., Lax N.Z., Turnbull D.M., Taylor R.W. (2017) The genetics and pathology of mitochondrial disease. J. Pathol. 241: 236–250. 27659608 10.1002/path.4809 PMC5215404

[cit0003] Anderson A.J., Jackson T.D., Stroud D.A., Stojanovski D. (2019) Mitochondria — hubs for regulating cellular biochemistry: emerging concepts and networks. Open Biol. 9: 190126. 31387448 10.1098/rsob.190126 PMC6731593

[cit0004] Bock F.J., Tait S.W.G. (2020) Mitochondria as multifaceted regulators of cell death. Nat. Rev. Mol. Cell Biol. 21: 85–100. 31636403 10.1038/s41580-019-0173-8

[cit0005] Bradford M.M. (1976) A rapid and sensitive method for the quantitation of microgram quantities of protein utilizing the principle of protein-dye binding. Anal. Biochem. 72: 248–254. 942051 10.1016/0003-2697(76)90527-3

[cit0006] Brocard J.B., Rintoul G.L., Reynolds I.J. (2003) New perspectives on mitochondrial morphology in cell function. Biol. Cell. 95: 239–242. 12941521 10.1016/s0248-4900(03)00062-5

[cit0007] Brown G.C., Borutaite V. (2007) Nitric oxide and mitochondrial respiration in the heart. Cardiovasc. Res. 75: 283–290. 17466959 10.1016/j.cardiores.2007.03.022

[cit0008] Brown G.C. (2010) Nitric oxide and neuronal death. Nitric Oxide 23: 153–165. 20547235 10.1016/j.niox.2010.06.001

[cit0009] Cao J.L., Adaniya S.M., Cypress M.W., Suzuki Y., Kusakari Y., Jhun B.S., O-Uchi J. (2019) Role of mitochondrial Ca^2+^ homeostasis in cardiac muscles. Arch. Biochem. Biophys. 663: 276–287. 30684463 10.1016/j.abb.2019.01.027 PMC6469710

[cit0010] Chen Y.-R., Zweier J.L. (2014) Cardiac mitochondria and reactive oxygen species generation. Circ. Res. 114: 524–237. 24481843 10.1161/CIRCRESAHA.114.300559 PMC4118662

[cit0011] Cherdal S., Mouline S., Amghar S. (2018a) SBGN2HFPN transformation of SBGN-PD into Petri nets illustrated on the glycolysis pathway. Int. J. Intel. Eng. Syst. 11: 275–289.

[cit0012] Cherdal S., Mouline S. (2018b) Modelling and simulation of biochemical processes using Petri nets. Processes 6: 97.

[cit0013] Cherenok S.O., Yushchenko O.A., Tanchuk V.Yu., Mischenko I.M., Samus N.V., Ruban O.V., Matvieiev Y.I., Karpenko J.A., Kukhar V.P., Vovk A.I., Kalchenko V.I. (2012) Calix[4]arene-α-hydroxyphosphonic acids. Synthesis, stereochemistry, and inhibition of glutathione S-transferase. Arkivoc. 4: 278–298.

[cit0014] Danylovych H.V., Danylovych Yu.V., Gulina M.O., Bohach T.V., Kosterin S.O. (2019) NO-synthase activity in the mitochondria of the uterus smooth muscle: identification and biochemical properties. Gen. Physiol. Biohys. 38: 39–50. 10.4149/gpb_2018034 30657455

[cit0015] Danylovych Y.V., Danylovych H.V., Kolomiets O.V., Sviatnenko M.D., Kosterin S.O. (2022) Biochemical properties of H^+^-Ca^2+^-exchanger in the myometrium mitochondria. Curr. Res. Physiol. 5: 369–380. 36176920 10.1016/j.crphys.2022.09.005 PMC9513619

[cit0016] Danylovych G.V., Kolomiets O.V., Danylovych Yu.V., Rodik R.V., Kalchenko V.I., Kosterin S.O. (2018) Calix[4]arene C-956 is effective inhibitor of H^+^-Ca^2+^-exchanger in smooth muscle mitochondria. Ukr. Biochem. J. 90: 25–31.

[cit0017] Demicheli V., Moreno D.M., Jara G.E., Lima A., Carballal S., Rios N., Batthyany C., Ferrer-Sueta G., Quijano C., Estrin D.A., Marti M.A., Radi R. (2016) Mechanism of the reaction of human manganese superoxide dismutase with peroxynitrite: nitration of critical tyrosine 34. Biochemistry 55: 3403–3417. 27227512 10.1021/acs.biochem.6b00045

[cit0018] Dunn J.-D., Alvarez L.A.J., Zhang X., Soldati T. (2015) Reactive oxygen species and mitochondria: a nexus of cellular homeostasis. Redox Biol. 6: 472–485. 26432659 10.1016/j.redox.2015.09.005 PMC4596921

[cit0019] Formanowicz D., Radom M., Zawierucha P., Formanowicz P. (2017) Petri net-based approach to modeling and analysis of selected aspects of the molecular regulation of angiogenesis. PLoS ONE. 12: e0173020. 28253310 10.1371/journal.pone.0173020 PMC5333880

[cit0020] Gellerich F.N., Gizatullina Z., Trumbeckaite S., Nguyen H.P., Pallas T., Arandarcikaite O., Vielhaber S., Seppet E., Striggow F. (2010) The regulation of OXPHOS by extramitochondrial calcium. Biochim. Biophys. Acta. 1797: 1018–1027. 20144582 10.1016/j.bbabio.2010.02.005

[cit0021] Giulivi C., Kato K., Cooper C.E. (2006) Nitric oxide regulation of mitochondrial oxygen consumtion I: cellular physiology. Am. J. Physiol. 291: C1225–C1231. 10.1152/ajpcell.00307.2006 16885394

[cit0022] Giulivi C. (2007) Mitochondria as generators and targets of nitric oxide. Novartis Found. Symp. 287: 92–104. 18074633

[cit0023] Gupta S., Fatima Z., Kumawat S. (2021) Study of the bioenergetics to identify the novel pathways as a drug target against Mycobacterium tuberculosis using Petri net. BioSystems 209: 104509. 34461147 10.1016/j.biosystems.2021.104509

[cit0024] Heikal A.A. (2010) Intracellular coenzymes as natural biomarkers for metabolic activities and mitochondrial anomalies. Biomarker Med. 4: 241–263. 10.2217/bmm.10.1 PMC2905054 20406068

[cit0025] Kaasik A., Safiulina D., Zharkovsky A., Veksler V. (2007) Regulation of mitochondrial matrix volume. Am. J. Physiol. Cell Physiol. 292: C157–C163. 16870828 10.1152/ajpcell.00272.2006

[cit0026] Keleti T. (1986) *Basic enzyme kinetics*. Budapest. Akademiai Kiado.

[cit0027] Kosterin P., Kim G.H., Muschol M., Obaid A.L., Salzberg B.M. (2005) Changes in FAD and NADH fluorescence in neurosecretory terminals ar triggered by calcium entry and by ADP production. J. Membr. Biol. 208: 113–124. 16645741 10.1007/s00232-005-0824-x

[cit0028] Litvinova L., Atochin D.N., Fattakhov N., Vasilenko M., Zatolokin P., Kirienkova E. (2015) Nitric oxide and mitochondria in metabolic syndrome. Front Physiol. 6: 20. 25741283 10.3389/fphys.2015.00020 PMC4330700

[cit0029] Matuz-Mares D., González-Andrade M., Araiza-Villanueva M.G., Vilchis-Landeros M.-M., Vázquez-Meza H. (2022) Mitochondrial calcium: effects of its imbalance in disease. Antioxidants 11: 801. 35624667 10.3390/antiox11050801 PMC9138001

[cit0030] Merkus H.G. (2009) *Particle size measurements. Fundamentals, practice, quality*. Springer.

[cit0031] Moon Y., Balke J.E., Madorma D., Siegel M.P., Knowels G., Brouckaert P., Buys E.S., Marcinek D.J., Percival J.M. (2017) Nitric oxide regulates skeletal muscle fatigue, fiber type, microtubule organization, and mitochondrial ATP synthesis efficiency through cGMP-dependent mechanisms. Antioxid. Redox Signal. 26: 966–985. 27393340 10.1089/ars.2016.6630 PMC5467110

[cit0032] Nowikovski K., Schweyen R.J., Bernardi P. (2009) Pathophysiology of mitochondrial volume homeostasis: potassium transport and permeability transition. Biochim. Biophys. Acta. 1787: 345–350. 19007745 10.1016/j.bbabio.2008.10.006

[cit0033] Pan Y.-C., Hu X.-Y., Guo D.-S. (2021) Biomedical applications of thiacalixarenes: state of the art and perspectives. Angewandte Chemie Int. Ed. 60: 2768–2794. 10.1002/anie.201916380 31965674

[cit0034] Piantadosi C.A., Suliman H.B. (2012) Redox regulation of mitochondrial biogenesis. Free Radic. Biol. Med. 53: 2043–2053. 23000245 10.1016/j.freeradbiomed.2012.09.014 PMC3604744

[cit0035] Salem M.M., Shalbaf M., Gibbons N.C., Chavan B., Thornton J.M., Schallreuter K.U. (2009) Enhanced DNA binding capacity on up-regulated epidermal wild-type p53 in vitiligo by H2O2-mediated oxidation: a possible repair mechanism for DNA damage. FASEB J. 23: 3790–3807. 19641144 10.1096/fj.09-132621

[cit0036] Santos C.X.C., Anilkumar N., Zhang M., Brewer A.C., Shah A.M. (2011) Redox signaling in cardiac myocytes. Free Radic. Biol. Med. 50: 777–793. 21236334 10.1016/j.freeradbiomed.2011.01.003 PMC3049876

[cit0037] Shiva S. (2010) Mitochondria as metabolizers and targets of nitrite. Nitric Oxide. 22: 64–74. 19788924 10.1016/j.niox.2009.09.002 PMC2819587

[cit0038] Tengan C.H., Rodrigues G.S., Godinho R.O. (2012) Nitric oxide in skeletal muscle: role on mitochondrial biogenesis and function. Int. J. Mol. Sci. 13: 17160–17184. 23242154 10.3390/ijms131217160 PMC3546744

[cit0039] Tortora V., Quijano C., Freeman B., Radi R., Castro L. (2007) Mitochondrial aconitase reaction with nitric oxide, S-nitrosoglutathione, and peroxynitrite: mechanisms and relative contributions to aconitase inactivation. Free Radic. Biol. Med. 42: 1075–1088. 17349934 10.1016/j.freeradbiomed.2007.01.007

[cit0040] Traaseth N., Elfering S., Solien J., Haynes V., Giulivi C. (2004) Role of calcium signaling in activation of mitochondrial nitric oxide synthase and citric acid cycle. Biochim. Biophys. Acta 1658: 64–71. 15282176 10.1016/j.bbabio.2004.04.015

[cit0041] Valdez L.B., Zaobornyj T., Boveris A. (2006) Mitochondrial metabolic states and membrane potential modulate mtNOS activity. Biochim. Biophys. Acta 1757: 166–172. 16624252 10.1016/j.bbabio.2006.02.013

[cit0042] Veklich T.O. (2016) The inhibitory influence of calix[4]arene of C-90 on the activity of Ca^2+^,Mg^2+^-ATPases in plasma membrane and sarcoplasmic reticulum in myometrium Fells. Ukr. Biochem. J. 88: 5–15. 10.15407/ubj88.02.005 29227596

[cit0043] Wingender E. (ed.) (2011) *Biological Petri Nets*. IOSPress.

[cit0044] Wray S., Prendergast C. (2019) The myometrium: from excitation to contractions and labour. Adv. Exp. Med. Biol. 1124: 233–263. 31183830 10.1007/978-981-13-5895-1_10

[cit0045] Yang W., Wang W., Guo R., Gong L., Gong S. (2012) Convenient direct syntheses of selectively para-substituted di-, tri- and tetra-formylated thiacalix[4]arenes. Eur. J. Org. Chem. 2012(17): 3326–3330.

[cit0046] Yanga T., Lina C., Fua H., Jianga Y., Zhao Y. (2004) An efficient method for synthesis of 4-(phosphonomethyl)benzene derivatives under solvent-free conditions. Synth. Commun. 34: 1017–1022.

